# Case report: Takotsubo syndrome following percutaneous coronary intervention

**DOI:** 10.1186/s13019-023-02412-0

**Published:** 2023-11-16

**Authors:** Rui Lu, Mingjun Lu, Shangfei He, Jing Lu, Yi Liao, Tongtao Cui, Min Wang

**Affiliations:** 1 Department of Vasculocardiology, The First Affiliate Hospital of Guang Zhou Medical University, Guangzhou, China; 2 Department of Neurology, The First Affiliated Hospital of Hainan Medical University, Haikou, China

**Keywords:** Takotsubo syndrome (TTS), Percutaneous coronary intervention (PCI), Differential diagnosis, Mental service

## Abstract

**Background:**

Takotsubo syndrome (TTS), which is frequently secondary to severe emotional (fear, anxiety, etc.) or physical stress, is an acute reversible heart failure syndrome characterized by temporary left ventricular regional systolic dysfunction. Nevertheless, TTS after percutaneous coronary intervention (PCI) is rare, and its clinical characteristics are easily confused with complications after PCI.

**Case presentation:**

This article reports a case of TTS induced by psychological and physical pressure after successful PCI in our institution. The patient had symptoms comparable to complications after PCI, including V1-V5 ST segment elevation and T wave changes of electrocardiogram (ECG) and troponin elevation. Coronary angiogram, left ventricle opacification (LVO), and cardiac magnetic resonance (CMR) were performed to exclude postoperative complications. Diagnosis of TTS was eventually achieved.

**Conclusion:**

We cannot dismiss the risk of TTS in patients who have unexplained V1-V5 ST segment elevation and T wave changes of ECG and troponin elevation following successful PCI. Meanwhile, medical personnel should provide mental, cultural, and emotional services to patients in addition to essential diagnostic and treatment technical services during the perioperative period.

## Background

TTS occurs normally in postmenopausal women, which is an acute reversible left ventricular dysfunction caused by intense emotional or physical stress. Clinical features include chest pain, ST segment elevation seen on ECG, elevation of cardiac biomarkers, and abnormal ventricular wall motion [1, 2]. An increase in the incidence of TTS appears to be a consequence of greater stress in modern life and growing knowledge of the disease among clinicians. The research found that there was a significant increase in the incidence of stress cardiomyopathy due to the COVID−19 pandemic. Before the pandemic, only between 1.5% and 1.8% of patients presenting with acute coronary syndrome were diagnosed with TTS, but 7.75% of patients in this population were diagnosed with TTS during the COVID−19 pandemic [3]. Additionally, there are many similarities between complications after PCI and TTS following PCI in clinical manifestations and auxiliary examinations, and both of them are often difficult to distinguish, which needs to be identified by coronary angiography and ventricular angiography. In this article, we reported one case of TTS induced by psychological and physical stress after PCI.

## Case presentation

**Fig. 1:**
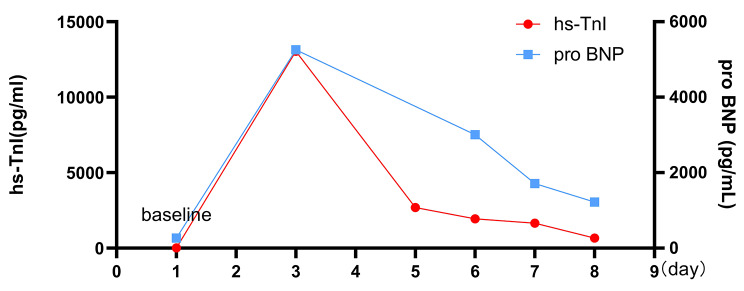
Hs-TnI and pro-BNP trend chart

**Fig. 2:**
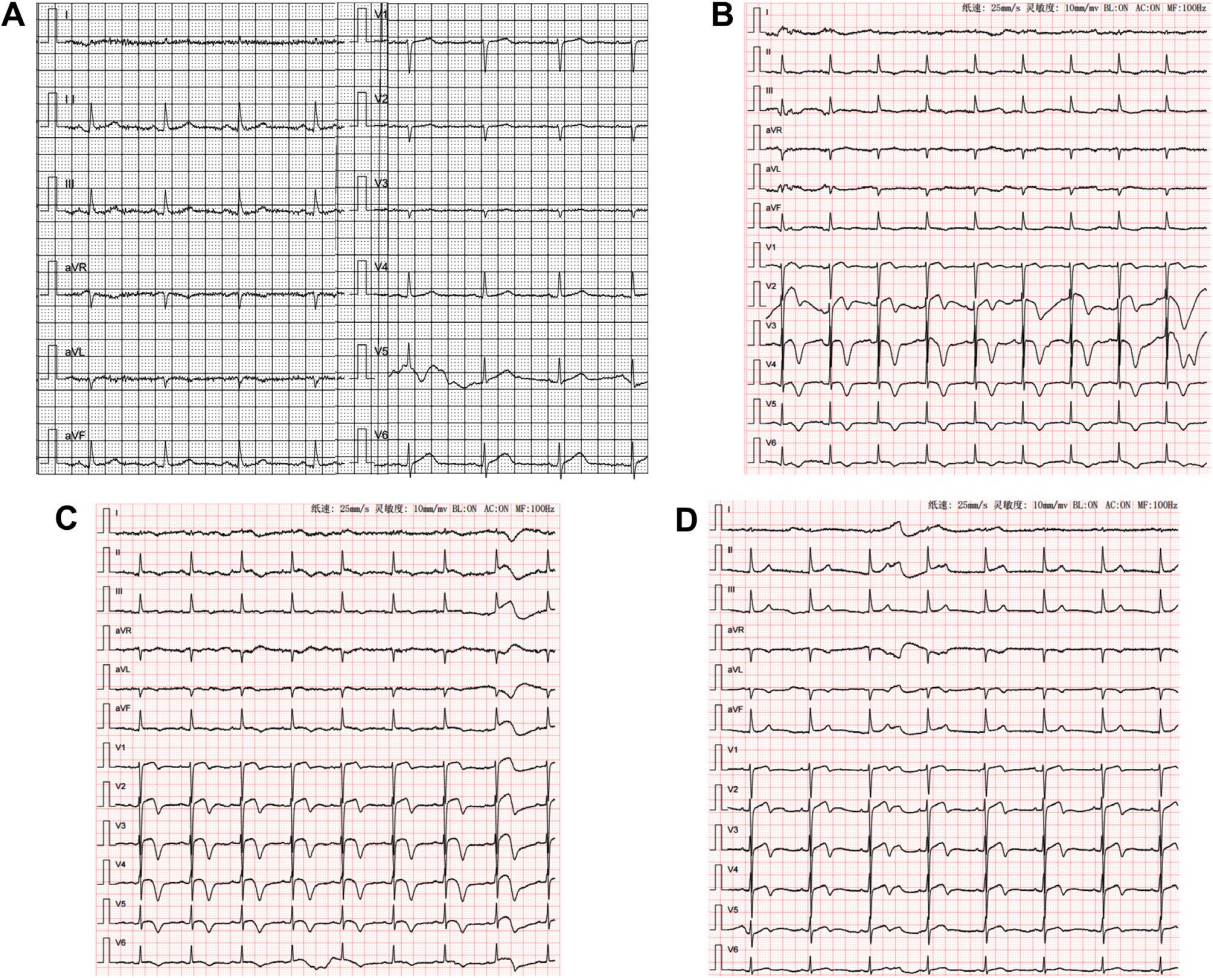
Dynamic ECG changes from admission to 7 days after PCI. (**A**) ECG on admission;(**B**) ECG on Day 1 after PCI; (**C**) ECG on Day 4 after PCI; (**D**) ECG on Day 7 after PCI.

**Fig. 3:**
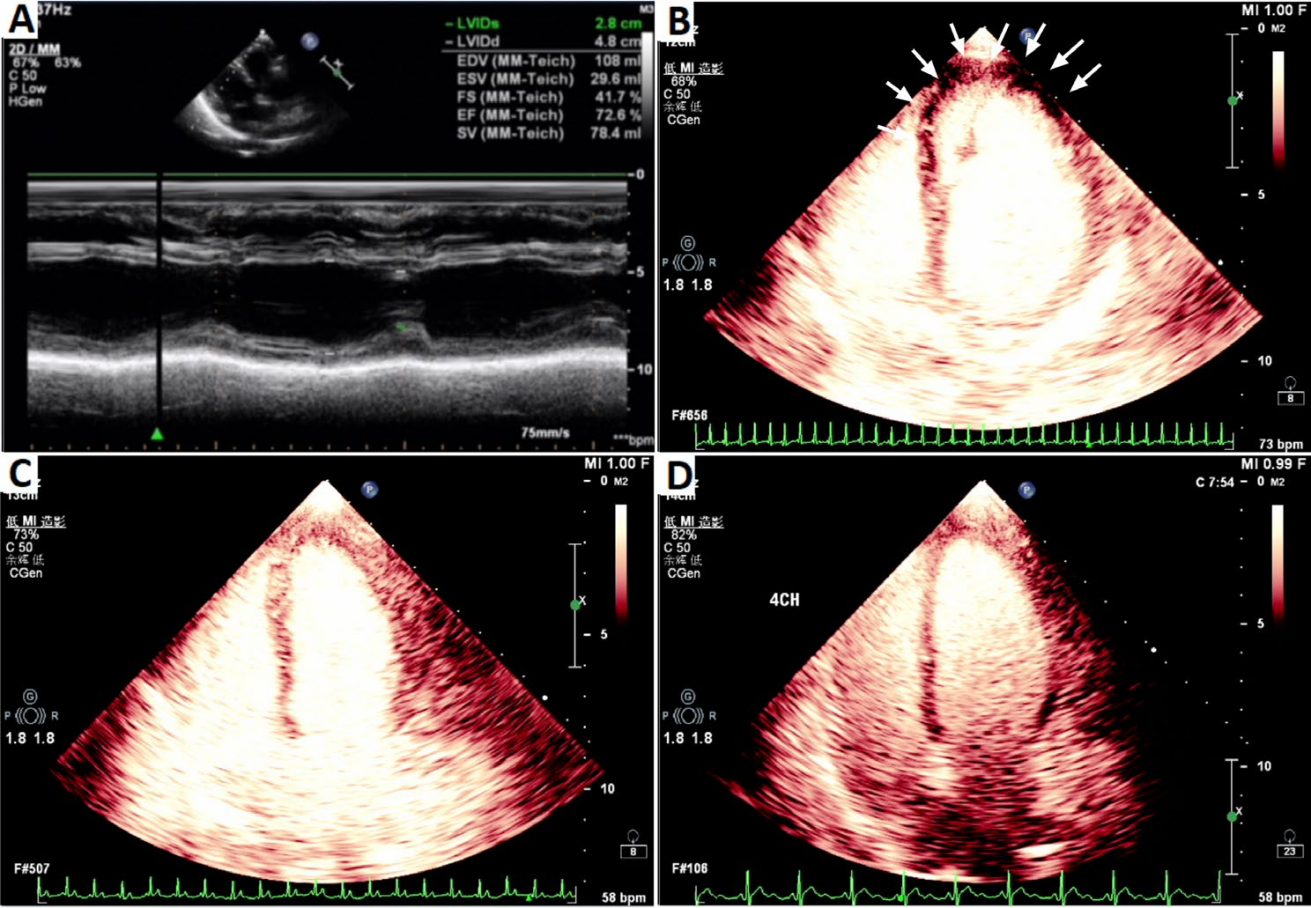
Normal echocardiography at admission (**A**). LVO on Day 1 after PCI shows the middle and lower segments of the ventricular septum, the anterior wall of the left ventricle, and the ventricular wall of the apical segment (arrow) at the onset of Takotsubo syndrome (**B**). On day 7 after PCI, only slight akinesia with a normal ejection fraction of 65% can be seen (**C**). Normal LVO at 2 months later (**D**)

**Fig. 4:**
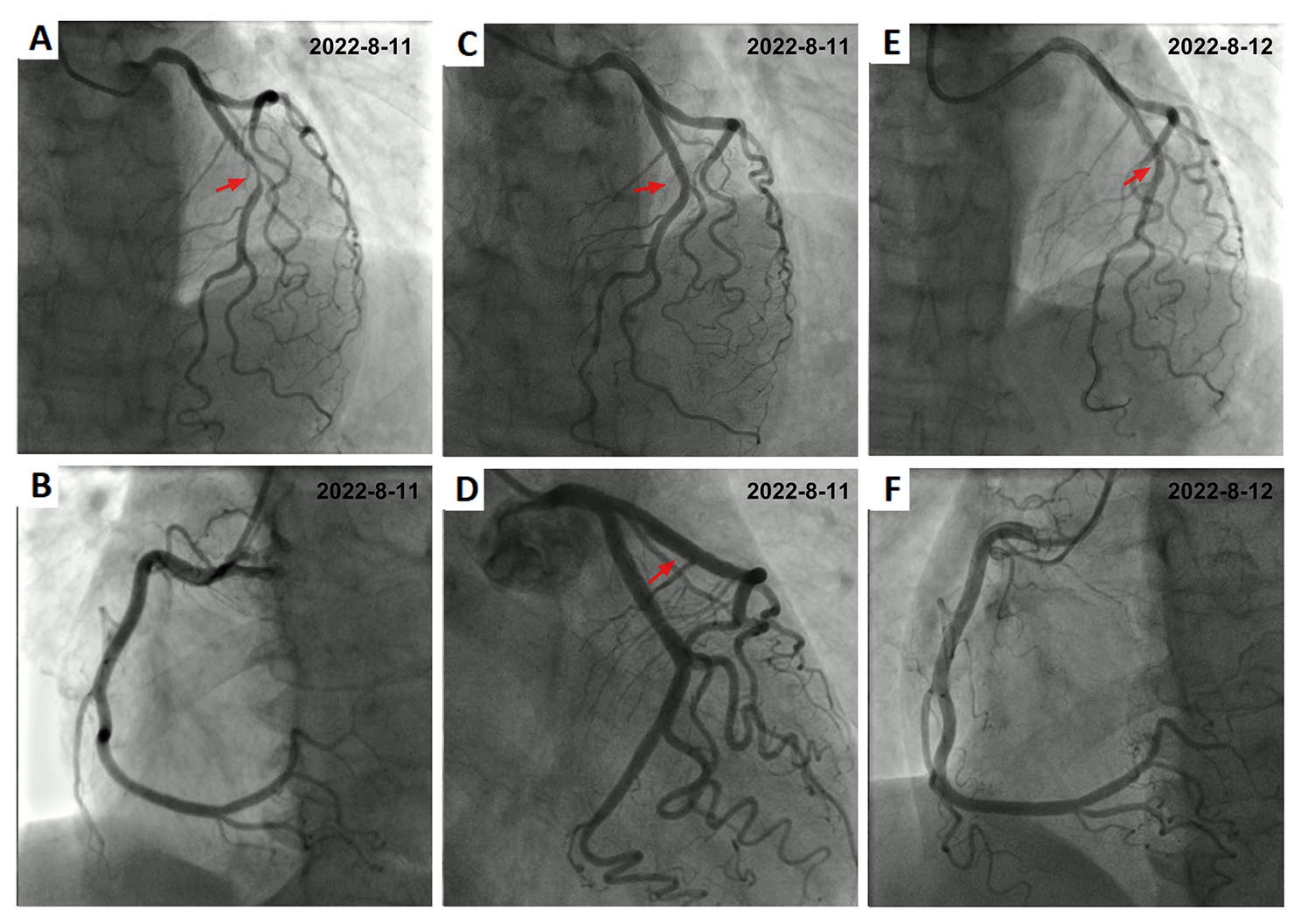
Coronary angiograms. (**A-B**) 3 coronary arteries before PCI. (**C-D**) Left coronary artery after PCI. (**E-F**) Re-examination of coronary angiogram following abnormal ECG.

A 69 years old female presented to our institution because of repeated episodes of chest tightness located over the area of the xiphoid process during exertion and at rest in the last week before presentation.Past medical history revealed erosive gastritis and no other chronic condition as hypertension, diabetes etc. The patient denies smoking or drinking habits. Under the current situation, her high sensitive troponin I (hs-TnI) level was 14.00pg/mL (normal value 0-17.5pg/mL), myoglobin level was 54.3ug/L (normal value < 70ug/L), Pro-B-type natriuretic peptide (Pro-BNP) level was 269.0pg/mL (normal value ≤ 300pg/mL) (Fig. 1), total cholesterol (TC) level was 5.98mmol/L (normal value 2.9-5.5mmol/L), low-density lipoprotein cholesterol (LDL-C) level was 3.85mmol/L (normal value 2.5-4.14mmol/L). The ECG showed sinus rhythm (Fig. 2A). Echocardiography revealed normal cardiac chamber size, the left ventricular ejection fraction (LVEF) was 73%(Fig. 3A). On the fourth day after admission, coronary angiogram showed that the bifurcation between the middle part of the left anterior descending artery (LAD) and the first diagonal branch (D1) of the LAD had a stenosis of about 80%. After accurate positioning, a 2.0 × 10 mm cutting balloon was used to dilate at 10 atmosphere (atm) pressure in the middle segment of the LAD, and a 2.5 × 15 mm hyperbaric balloon was used to dilate at 10–14 atm in lesions of the D1 bifurcation. Then, an Firebird 3.0 × 14 mm stent was implanted in the middle segment of the LAD. Then, a 2.0 × 15 mm hyperbaric balloon was delivered to the stent and post-dilated with 14–16 atm (avoid D1 bifurcation). There was no residual stenosis, no dissection or tear (Fig. 4A-D). TIMI III flow in Diagonal branch and the opening has improved compared to the basic state. The procedure went smoothly without signs of vascular entrapment, stent strut malapposition and tissue prolapse. The hs-Tnl value measured immediately after the intervention was 106.2pg/ml, and Tirofiban was continuously pumped in conjunction with oral antiplatelet drugs: 90 mg of Ticagrelor twice a day and 100 mg of Indobufen twice a day.

**Fig. 5:**
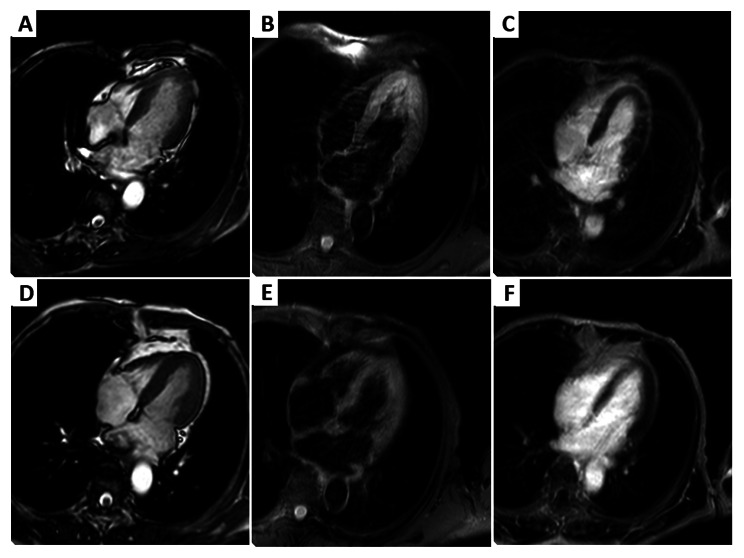
Comparison of CMR on Day 4 after PCI (**A-C**) and at 2 months follow-up (**D-F**). Before treatment, B-TEF four chamber showed the middle segments of the ventricular septum and left ventricular apex area are irregular in shape, with bulge of the regional area (**A**). T2-SPIR showed slightly increased signal intensity with edema in the left ventricular apex area (**B**). PSIR-TFE four chamber showed no late gadolinium enhancement. After treatment, these abnormalities were resolved (**D-F**).

On the first day after intervention, the patient developed chest tightness, the hs-TnI value was 13045.50pg/mL, the Pro-BNP was 5255.0pg/mL (Fig. 1), ECG showed sinus rhythm, ST segment elevation in leads V1-V5, T wave changes (Fig. 2B), emergency coronary angiogram showed stent shadow in the middle of LAD, and blood circulation in the stent was smooth. There is no obvious stenosis in the left circumflex coronary artery (LCX) and the right coronary artery (RCA) (Fig. 4E-F), and the blood flow in all three vessels is TIMI grade 3. LVO reveals that the middle and basal segments of the ventricular septum, the anterior wall of the left ventricle, and the ventricular wall of the apical segment become thinner, and the movement is hypokinesia with decreased perfusion, with LVEF of 51% (Fig. 3B). Subsequent CMR presented that middle and basal segments of the ventricular septum and the apex were severely hypokinesia (Fig. 5A-C).

Based on the patient’s medical history, symptoms, laboratory examination, imaging and other auxiliary examination results, the diagnosis is considered as (1) Takotsubo syndrome (TTS); (2) Coronary atherosclerotic heart disease. We initiated treatment with furosemide 60 mg/d、bisoprolol 2.5 mg/d as TTS pharmacological therapies. Simultaneously, as the patient had recently received a stent, she continued to be treated with dual antiplatelet drugs and statins. On the 4th day after coronary angioplasty, the ECG revealed inverted, broad, and deep T waves in leads V2~V4 was (Fig. 2C), and the levels of hs-TnI and Pro-BNP decreased significantly (Fig. 1). On the seventh postoperative day, LVO revealed great improvement of the left ventricular wall motion, with good myocardial perfusion and a LVEF of 65% (Fig. 3C). The patient was discharged after receiving an additional 12.5 mg of sacubitril/valsartan twice day.

Two months later, the patient returned to the institution for follow up and she had a hs-cTnI level of 12.30pg/mL, a pro-BNP level of 324.00pg/mL, and no evidence of ST-T segment changes on ECG. LVO demonstrated no obvious motion abnormalities in the myocardial segments of each ventricular wall, and the perfusion was adequate, with LVEF of 59% (Fig. 3D). CMR demonstrated improvement in left ventricular systolic function compared to the previous examination (Fig. 5D-F). Changes of echocardiography was summarized in Table 1.

**Table 1:** Changes of echocardiography

	At admission	Day 1 after PCI	Day 7 after PCI	2 months later
LAs (mm)	24	31	28	32
LVDd (mm)	48	40	37	45
LVDs (mm)	28	26	22	28
LVEF (%)	73	51	65	59

## Discussion

The pathophysiological mechanism of TTS remains unclear, but a significant body of research indicates that sympathetic overactivity and catecholamine surge are the most essential pathological mechanism of patho genesis [1, 4]. Acute physical or emotional triggers make sympathetic nervous system overstimulation, resulting in large increases in norepinephrine and epinephrine. At the same time, cortisol and catecholamine bioavailability will increase [5]. A study on animal models shows that a high intravenous epinephrine produces the characteristic reversible apical depression of myocardial contraction coupled with basal hypercontractility [6]. In recent years, it has been hypothesized that the distinctive aberrant apical movement in TTS is caused by the influence of excessive levels of epinephrine on cardiac G protein [7].

The patient in this case is a postmenopausal female. Several illnesses can explain her clinical symptoms following PCI, so distinguishing between them is critical (Table 2). First, the intervention was a success, with no coronary artery dissection, incomplete stent apposition, tissue prolapse, or other complications. Moreover, she was given anticoagulants and antiplatelet agents after the intervention, so postoperative complications (such as coronary artery dissection, stent thrombosis and coronary artery perforation) were unlikely to occur [8]. Second, repeated coronary angiography did not detect neither coronary artery dissection, nor stent thrombosis. Third, abnormalities in ventricular wall motion caused by coronary artery dissection or stent thrombosis are typically restricted to locations innervated by the culprit coronary artery. This patient’s ECG, echocardiography, and CMR show that the distribution of abnormal ventricular wall motion exceeds the area supplied by the left anterior descending artery. Last but not least, all clinical characteristics of the patient can be explained using TTS alone for the following reasons.

**Table 2:** Differential diagnosis between TTS and Complications after PCI.

	Takotsubo syndrome	Complications after PCI (coronary-artery dissection, stent thrombosis)
Symptoms	heart failure is the primary symptom. Chest tightness can occur, but it is usually mild, with less chest pain, nausea, and vomiting.	Severe chest pain, chest tightness, palpitations, nausea, and vomiting are common symptoms.
ECG	ECG shows ST segment elevation or T wave alterations that surpass the blood supply area of a single artery and changes dynamically as the disease progresses.	ST-T changes in the leads corresponding to the occluded vessel’s blood supply area, which changes dynamically as the disease progresses.
Echocardiography	The extent of ventricular wall motion abnormalities usually exceeds the area supplied by a single coronary artery,accompanied by abnormal myocardial perfusion.	The extent of ventricular wall motion abnormalities usually corresponds to the area supplied by a single coronary artery.
CMR	The aberrant range of ventricular wall motion typically surpasses the area dominated by a single coronary artery, and the severity of local myocardial edema, perfusion deficiency, and delayed enhancement varies significantly.	The area dominated by a single coronary artery usually corresponds to the aberrant range of ventricular wall motion. Local myocardial edema, perfusion deficit, and delayed enhancement are usually significant.
Operation	The intervention went without a hitch.	There may be factors such as the formation of vascular wall dissection, poor stent attachment, and tissue prolapse during the intervention.
Coronary angiograms	All 3 coronary arteries are smooth and the blood flow is unobstructed, no thrombus shadow and no contrast agent retention.	Vascular occlusion, thrombosis and contrast agent retention may be seen.

The patient’s education level is primary school. She has insufficient awareness of primary disease as a result of having difficult communicating with her doctors, limited knowledge and information, and lack of medical expertise during perioperative time, resulting in negative feelings such as anxiety and fear. Meanwhile, PCI is an invasive treatment that enhanced sympathetic activity and catecholamine release throughout the body. As a result, patients are under emotional and physical distress. Moreover, the ECG showed ST-segment elevation in leads V1-V5 and biphasic T-wave, and blood chemistry showed a significant rise in hs-TnI on the first post-procedure day. Nonetheless, emergency coronary angiography revealed that the blood flow at the stent implantation site was normal. At the same time, echocardiography and CMR revealed that the contractility of the middle and basal segments of the left ventricular septum and of the apex, was decreased, and the patient’s heart function improved rapidly after short-term symptomatic treatment. Therefore, the patient could be diagnosed with TTS based on the 2018 TTS international consensus [1]. This case highlights the difficulty of TTS diagnostic after PCI and the necessity of coronary angiography, echocardiography, and CMR for its differential diagnosis.

Finally, TTS has been regarded as a relatively benign condition with a generally favorable prognosis since its first description. Recent investigation, however, has indicated that patients experienced substantial mortality and morbidity following the acute phase of TTS, with all-cause mortality at 1 year being 5.6% [9]. Therefore, early clinical identification will aid in the development of individualized treatment strategies and clinical management. The primary goal of treating heart failure in TTS patients is to relieve pulmonary congestion and provide hemodynamic support [10, 11]. Treatment of TTS patients differs significantly depending on whether the left ventricular outflow pathway is obstructed or not. We used furosemide to control volume load and beta-blockers to reduce excessive myocardial contraction, decrease left ventricular filling, reduce heart rate, and improve cardiac remodeling in this TTS patient who developed acute heart failure without the left ventricular outflow tract obstruction during the acute phase. A number of randomized clinical trials have indicated that sacubitril/valsartan reduces renal and cardiac adverse events in patients with heart failure with preserved ejection fraction when compared to valsartan alone [12, 13]. Consequently, we began treatment with sacubitril valsartan sodium tablets on the 7th day after PCI. After a follow-up period of 2 mouths the patient was free of cardiac symptoms with a good functional capacity. The left ventricular ejection fraction appears normal.

## Conclusion

To summarize, we exclude other diseases, and diagnosis of TTS was eventually achieved, and the combined impact of emotional and physical stressors contribute to the incidence of TTS. This case also reminds us that when patients experience unexplained electrocardiogram changes and elevated troponin after PCI, we cannot ignore the possibility of TTS while considering the complications after PCI. Additionally, TTS caused by emotional stimulation is not rare, especially under the influence of negative emotional events [14–16]. In the perioperative period, patients receiving interventional treatment, should be provided a good physician to patient communication. As doctors, we should value the entire medical procedure,including not only providing patients with appropriate diagnostic and therapeutic technical services, but also timely explain the changes in the condition to patients and their families and properly removing the anxiety and fear caused by intervention. These not only improve the postoperative rehabilitation but also lower the incidence rate of TTS.

## Data Availability

The data underlying this article will be shared upon reasonable request to the corresponding author.
